# Multimodal Metabolic Imaging Reveals Pigment Reduction and Lipid Accumulation in Metastatic Melanoma

**DOI:** 10.34133/2021/9860123

**Published:** 2021-10-08

**Authors:** Hyeon Jeong Lee, Zhicong Chen, Marianne Collard, Fukai Chen, Jiaji G. Chen, Muzhou Wu, Rhoda M. Alani, Ji-Xin Cheng

**Affiliations:** ^1^Photonics Center, Department of Electrical and Computer Engineering, Department of Biomedical Engineering, Boston University, Boston, MA 02215, USA; ^2^Department of Dermatology, Boston University School of Medicine, Boston, MA 02118, USA

## Abstract

*Objective and Impact Statement*. Molecular signatures are needed for early diagnosis and improved treatment of metastatic melanoma. By high-resolution multimodal chemical imaging of human melanoma samples, we identify a metabolic reprogramming from pigmentation to lipid droplet (LD) accumulation in metastatic melanoma. *Introduction*. Metabolic plasticity promotes cancer survival and metastasis, which promises to serve as a prognostic marker and/or therapeutic target. However, identifying metabolic alterations has been challenged by difficulties in mapping localized metabolites with high spatial resolution. *Methods*. We developed a multimodal stimulated Raman scattering and pump-probe imaging platform. By time-domain measurement and phasor analysis, our platform allows simultaneous mapping of lipids and pigments at a subcellular level. Furthermore, we identify the sources of these metabolic signatures by tracking deuterium metabolites at a subcellular level. By validation with mass spectrometry, a specific fatty acid desaturase pathway was identified. *Results*. We identified metabolic reprogramming from a pigment-containing phenotype in low-grade melanoma to an LD-rich phenotype in metastatic melanoma. The LDs contain high levels of cholesteryl ester and unsaturated fatty acids. Elevated fatty acid uptake, but not *de novo* lipogenesis, contributes to the LD-rich phenotype. Monounsaturated sapienate, mediated by FADS2, is identified as an essential fatty acid that promotes cancer migration. Blocking such metabolic signatures effectively suppresses the migration capacity both *in vitro* and *in vivo*. *Conclusion*. By multimodal spectroscopic imaging and lipidomic analysis, the current study reveals lipid accumulation, mediated by fatty acid uptake, as a metabolic signature that can be harnessed for early diagnosis and improved treatment of metastatic melanoma.

## 1. Introduction

Melanoma is the most aggressive form of skin cancer. When melanoma is caught in its early stages and surgically removed, the prognosis is favorable; once melanoma has metastasized, it becomes difficult to treat [1, 2]. Although increasing Breslow depth, or melanoma tumor thickness, is associated with worse survival [3], the majority of deaths attributed to melanoma are due to thin tumors (<1 mm) [4]. With the incidence of melanoma steadily rising at a rate of 3% per year [5], it is more important than ever to detect melanoma at an early stage and identify the tumors that are the most at-risk for metastasizing for early intervention. Nevertheless, without molecular signatures, precision diagnosis and treatment of metastatic melanoma remain difficult.

One promising target is melanin, the major pigment in melanoma. It was found that more eumelanin than pheomelanin is present in malignant melanoma compared to dysplastic nevi [6, 7]. A recent study used pump-probe microscopy as an elegant label-free approach to distinguish the two types of melanin in melanoma tissues [8], heralding potential prognostic significance [9]. Despite this advance, loss of microphthalmia-associated transcription factor (MITF), the gene regulating pigmentation, is reported in melanomas with an invasive phenotype [10, 11], indicating an unmet need to identify new molecular markers for detecting aggressive and invasive melanoma.

Melanoma exhibits a dynamic and adaptive phenotypic switching capability [12]. Among the signaling pathways mediating this process, the MITF status is considered a central regulator of the phenotypic switch [13], namely, from a highly proliferative, less invasive to a less proliferative, highly invasive state. Specifically, loss of MITF is an indication of dedifferentiation of melanoma [14, 15], which is supported by enhanced migration/invasion gene expression profiles [16]. Despite these advances in the genetic characterization of melanoma phenotypes, metabolic markers for advanced melanoma have just begun to be explored. A recent report suggested elevated lipid uptake in metastatic melanoma [17], indicating that cancer progression is partly sustained by high lipogenic or lipid uptake activity; however, due to difficulties in compositional analysis, the exact metabolites and precise mechanisms that regulate melanoma progression remain unknown.

Here, we report findings of metabolic reprogramming in metastatic melanoma made by a multimodal chemical imaging platform. Specifically, by coregistered stimulated Raman scattering (SRS) imaging of biomolecules [18, 19] and pump-probe imaging of pigments [20] at a subcellular level, we revealed a previously unrecognized metabolic reprogramming from pigmentation in low-grade melanoma to lipid droplet (LD) accumulation in metastatic melanoma. Raman spectroscopy and lipidomic analysis further identified a significant amount of unsaturated fatty acids and cholesteryl ester (CE) in these LDs. Blocking fatty acid uptake and depleting LDs dramatically suppresses cell migration. In particular, the unsaturated fatty acid, sapienate, synthesized through fatty acid desaturase 2 (FADS2), is found to play an essential role in promoting melanoma migration, which may be mediated through membrane fluidity regulation. Consequently, inhibition of FADS2 suppresses melanoma migration *in vitro* and metastasis *in vivo*. In parallel, inhibiting cholesterol esterification is found to significantly reduce LD accumulation and suppress cell migration by inactivation of the Wnt/*β*-catenin pathway. Together, these results present a unique metabolic reprogramming profile that can be used for molecular diagnosis and treatment of metastatic melanoma.

## 2. Results

### 2.1. Melanoma Progression into Metastatic State Is Accompanied by Metabolic Reprogramming from Pigmentation to Lipid Accumulation

**Figure 1:**
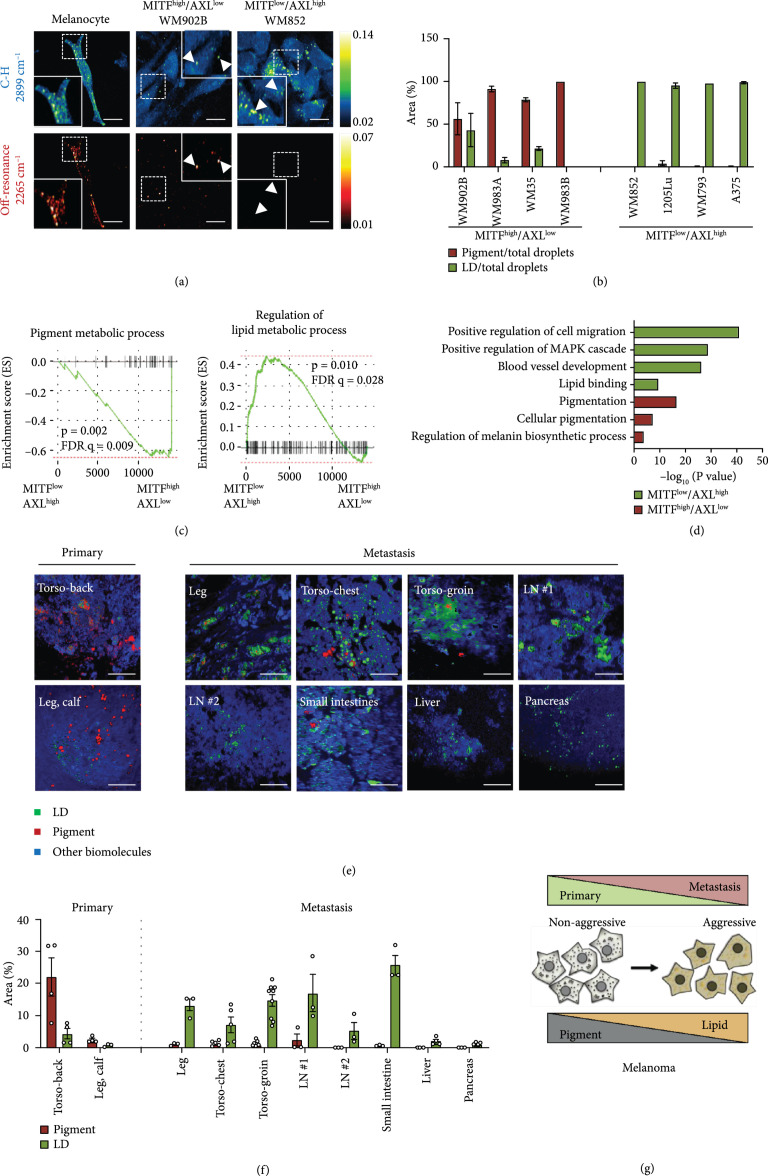
Metastatic melanoma exhibits an LD-rich phenotype. (a) Representative SRS images of human melanoma cells in the C-H region (2899 cm^-1^) and in the off-resonance region (2265 cm^-1^). Arrows indicate droplets. Scale bars, 20 *μ*m. (b) Quantification of droplets identified as pigment and LD from phasor analysis. n=4-6 fields of view. (c) GSEA of human melanoma samples from TCGA-SKMC based on MITF and AXL expression profiles. (d) Functional enrichment network analysis for MITF^low^/AXL^high^ melanoma and MITF^high^/AXL^low^ melanoma. (e) Multimodal imaging and phasor output of patient primary and metastatic melanoma tissue samples. LN: lymph node; SI: small intestines. Scale bars, 50 *μ*m. (f) Percent area of pigment and LD presented in the tissue. n=3-9 fields of view. (g) Metabolic reprogramming observed during melanoma progression from primary to metastasis. Data represent mean±standard error of the mean (SEM).

To investigate the metabolic reprogramming during melanoma progression, human melanoma cell lines bearing high MITF or low MITF expression were used. Consistent with previous reports [11], the melanoma cells with high MITF expression levels exhibit low AXL Receptor Tyrosine Kinase (AXL) expression (Figure S1a, b). On the basis of this expression profile, the human melanoma cell lines used in this study (Table S1) were clustered into MITF^high^/AXL^low^ or MITF^low^/AXL^high^ groups. To map the various metabolites at sub-cellular resolution in human melanoma cells, we deployed stimulated Raman scattering microscopy [18, 19]. The laser beating frequency was initially tuned to be resonant with C-H stretching vibration at 2899 cm^-1^. We detected a stimulated Raman loss signal arising from C-H-rich biomolecules, showing a clear cell morphology with a number of droplets in melanocytes and MITF^high^/AXL^low^ and MITF^low^/AXL^high^ melanoma cells (Figure 1(a)). These droplet structures in an SRS image are often considered to be lipid droplets (LDs) [18]. To verify the chemical specificity, we tuned the laser beating frequency to 2265 cm^-1^, off-resonant with C-H stretching vibration. No signal was observed from the off-resonance image of MITF^low^/AXL^high^ melanoma cells, which suggests that these droplets are rich in C-H bonds and are likely LDs (Figure 1(a)). This assignment was confirmed by immunofluorescence staining of an LD-associated protein, adipophilin (Figure S1c).

Intriguingly, droplets with strong signals were still observed off the Raman resonance in melanocytes and MITF^high^/AXL^low^ melanoma, indicating that signals from these droplets are not specific to C-H stretching vibration (Figure 1(a)). We attribute the strong signal at off-Raman resonance to either transient absorption or photothermal effects, which are often detected with a pump-probe imaging scheme [21]. To verify this hypothesis, we performed a time-resolved measurement. The signals from the droplets in MITF^high^/AXL^low^ melanoma show a fast decay within 1.0 picosecond and then remained at the same level (Figure S1d). The fast decay and the remaining signal are attributed to transient absorption and photothermal processes, respectively [22]. In comparison, the pump-probe decay curve of eumelanin powder extracted from *Sepia officinalis* showed a similar profile (Figure S1e). Unlike the pigments in melanoma, the pigments from melanocytes exhibit a negative decay curve (Figure S1d), indicating a different type of pigment in melanocytes. It was reported that when imaged under a pump-probe microscope, pheomelanin exhibits a negative signal whereas eumelanin exhibits a positive signal [8]. Thus, it is likely that the pigments in melanoma are mostly eumelanin and the pigments in melanocytes are mostly pheomelanin. Consistent with this observation, it was reported that eumelanin is presented in higher content in melanoma compared to nonmalignant nevi [6, 8]. Collectively, these results support that both melanocytes and MITF^high^/AXL^low^ melanoma cells have high pigmentation activity, concordant with high MITF expression.

To establish a method for quantifying pigmentation and LD accumulation in melanoma, we designed a time-domain multimodal SRS/pump-probe imaging and phasor analysis approach (Figure S2). In SRS imaging, 802 nm serves as a pump beam and 1045 nm serves as a Stokes beam, while in pump-probe imaging, 1045 nm serves as a pump beam and 802 nm serves as a probe beam (Figure S2a). SRS and pump-probe signals show different profiles in the time-domain: SRS displays a sharp Gaussian-shape peak, while pump-probe displays a slow decay curve (Figure S2b, c). These two signals can be well-separated by phasor analysis of the time-resolved measurements [23] (Figure S2d). Furthermore, we found that the subtle SRS spectral difference between LDs versus the rest of the cell can be further separated through phasor analysis (Figure S2d). Therefore, using the multimodal imaging and phasor analysis approach, we simultaneously imaged and quantified LD accumulation and pigmentation in human melanoma cell lines (Figure 1(b) and Figure S2e, f). There is a high percentage of pigment droplets in MITF^high^/AXL^low^ melanoma. Nevertheless, almost all droplets identified in MITF^low^/AXL^high^ melanoma are LDs (Figure 1(b)). Together, these data indicate a reduction of pigmentation and increase of lipid accumulation during the progression of melanoma from low grade to high grade.

Consistent with the metabolic profile observed in the cell lines, the Gene Set Enrichment Analysis (GSEA) of human melanoma samples from The Cancer Genome Atlas (TCGA) showed that the pigment metabolic process gene set was enriched in the MITF^high^/AXL^low^ group, while regulation of the lipid metabolic process gene set was enriched in the MITF^low^/AXL^high^ group (Figure 1(c) and Table 1(c) and Table S2). Differentially expressed genes (DEGs) were generated based on the median expression level of MITF and AXL (Figure S3a and Table S3). Functional enrichment network analysis for MITF^low^/AXL^high^ melanoma identified several gene sets related to aspects of cancer metastasis, such as cell migration and blood vessel development and lipid binding processes that were enriched from the upregulated genes, while gene sets related to pigmentation were enriched from the downregulated genes (Figure 1(d), Figure S3b, and Table S3). These results indicate a distinct metabolic profile of MITF^high^/AXL^low^ versus MITF^low^/AXL^high^ melanoma.

To validate the clinical relevance of such metabolic reprogramming in metastatic melanoma, we performed multimodal imaging of two primary and eight metastatic melanoma tissues from deidentified human melanoma patients (Figure 1(e)). The tumor lesions were confirmed by pathological assessment of the neighboring slices stained with hematoxylin and eosin (Figure S4). By time-resolved multimodal imaging and phasor analysis, signals from pigment, LDs, and other biomolecules were separated and quantified (Figures 1(e) and 1(f)). Primary melanoma tissues contain higher pigment content than LD content. In contrast, metastatic melanomas, including metastatic lesions on the extreme leg, torso-chest, and torso-groin, and metastatic tumors in other organs such as the lymph nodes, small intestines, liver, and pancreas, contain higher LD content than pigment content (Figure 1(f)). These results collectively suggest a metabolic reprogramming from pigmentation to lipid accumulation during the progression of human melanoma from the primary tumor to the metastatic tumor (Figure 1(g)).

### 2.2. The LDs in MITF^low^/AXL^high^ Melanoma Contain CE and Unsaturated Fatty Acids

**Figure 2:**
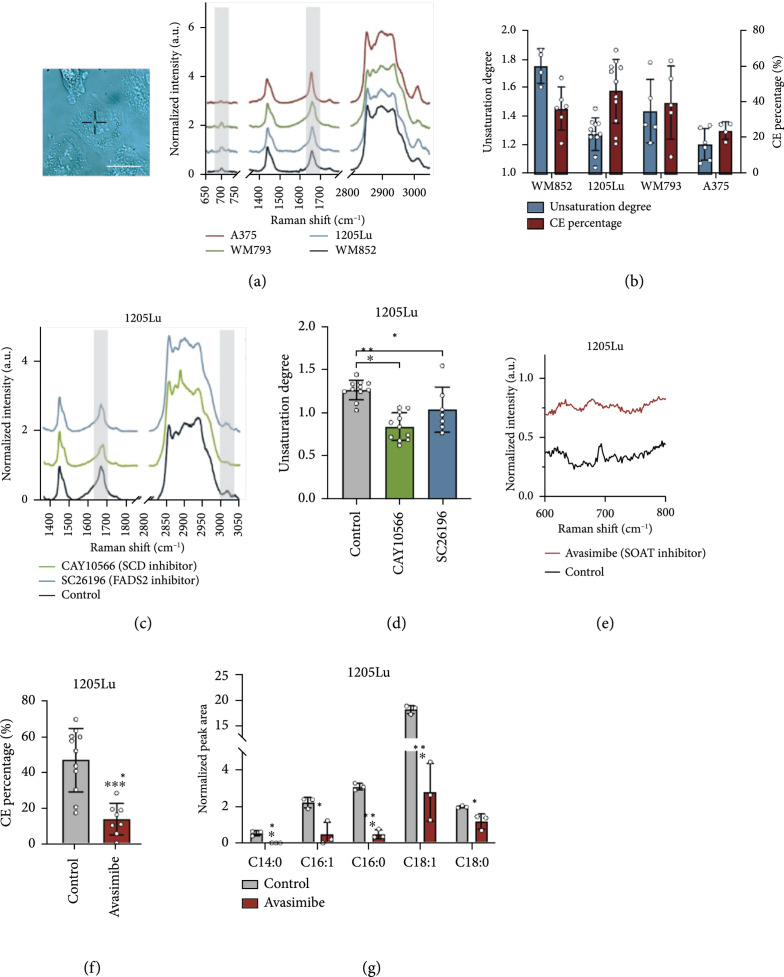
LDs in aggressive melanoma contain unsaturated fatty acids and CE. (a) Representative Raman spectra of single LDs in MITF^low^/AXL^high^ melanoma. The spectral intensity was normalized by the peak at 1445 cm^-1^. Grey areas highlight the Raman peaks used to quantify unsaturation degree and CE percentage. Insert shows the brightfield image of single LD identified in a cell. Scale bar: 10 *μ*m. (b) Quantification of unsaturation degree and CE percentage based on the calibration curves. n=4-11. (c) Representative Raman spectra of LDs in 1205Lu treated with DMSO as control, CAY10566 (50 *μ*M, 2 days), and SC26196 (50 *μ*M, 2 days). Grey areas highlight the Raman peaks indicative of unsaturation lipids. (d) Quantification of unsaturation degree calculated from the calibration curve. n=7-11. (e) Representative Raman spectra of LDs in 1205Lu treated with DMSO as control and avasimibe (10 *μ*M, 2 days). (f) Quantification of CE percentage in LDs calculated from the calibration curve. n=8-11. (g) Quantification of LC/MS measurement of lipids extracted from control and avasimibe-treated 1205Lu cells (10 *μ*M, 2 days). n=3 biological replicates. Data represent mean±SD. ${}^{*}p<0.05$, ${}^{**}p<0.01$, and ${}^{***}p<0.001$.

To unveil the composition of LDs accumulated in MITF^low^/AXL^high^ melanoma, we performed confocal Raman spectroscopic measurement of single LDs (Figure 2(a)). The Raman spectra of LDs in all MITF^low^/AXL^high^ melanoma cells showed similar spectral profiles, including bands for lipids in the fingerprint region (1200-1800 cm^-1^) and prominent CH_2_ stretching band (2850 cm^-1^). More detailed spectral analysis revealed that these LDs show peaks from the C=C stretching vibration at 1654 cm^-1^ and the vibration of =C-H bonds at 3002 cm^-1^, both suggesting the presence of unsaturated fatty acids (Figure 2(a)). To quantify the unsaturation degree, we generated a calibration curve using fatty acids containing various numbers of C=C bonds (Figure S5a). Raman spectra of palmitate (no C=C bond), oleate (one C=C bond), and linoleate (two C=C bonds) were obtained and analyzed. The peak intensity of C=C stretching vibration at 1654 cm^-1^ is found to be linearly correlated with the number of C=C bonds present in the fatty acids after normalization by the CH_2_ bending band at 1445 cm^-1^ (Figure S5a, b). On the basis of this calibration, we estimated the unsaturation degree to be 1.2 in WM852, 1.3 in 1205Lu, 1.4 in WM793, and 1.8 in A375 LD-rich cells (Figure 2(b)). To validate the presence of unsaturated fatty acids in LDs, we treated LD-rich melanoma cells with desaturase inhibitors, CAY10566 and SC26196, for inhibiting Stearoyl-CoA desaturase-1 (SCD) and FADS2, respectively. Cells treated with either the SCD or FADS2 inhibitor showed reduced peak intensities at both 1654 cm^-1^ and 3002 cm^-1^ (Figure 2(c)), resulting in the unsaturation degree of below or close to 1.0 (Figure 2(d)). Furthermore, the multiple reaction monitoring (MRM) profiling of neutral lipids from MITF^low^/AXL^high^ melanoma cells shows a high level of monounsaturated fatty acids in triglycerides (Figure S5c). Together, these results indicate that the LDs accumulated in MITF^low^/AXL^high^ melanoma contain a significant amount of unsaturated fatty acids.

Another important component that we identified from the Raman spectral profile is cholesteryl ester (CE). This component was indicated by the cholesterol band at 702 cm^-1^ and the C=O ester stretching band at 1740 cm^-1^ (Figure 2(a)). To quantify the CE percentage, we generated calibration curves for CE percentage using CE and triacylglycerol emulsions (Figure S5d, e). The Raman spectra of lipid emulsions with various ratios of CE and triacylglycerol showed that the peak intensity of the cholesterol band at 702 cm^-1^ was linearly correlated to the molar percentage of CE in the lipid emulsions after normalization by the CH_2_ bending band at 1445 cm^-1^ (Figure S5d, e). On the basis of the calibration, it was estimated that the CE contents of the LDs are 37.3%, 47.3%, 40.6%, and 25.0% in WM852, 1205Lu, WM793, and A375, respectively (Figure 2(b)). Lipidomic analysis by MRM profiling shows similar CE percentage in neutral lipids (Figure S5f). Inside cells, excess cholesterol is known to be esterified by the sterol O-acyltransferase (SOAT) enzyme and stored in LDs [24]. To validate that CE is indeed stored in LDs, we treated cells with a potent SOAT inhibitor, avasimibe. After avasimibe treatment, the peak intensity at 702 cm^-1^ reduced significantly (Figure 2(e)), indicating less than 20% CE in the LDs (Figure 2(f)). Furthermore, the LC/MS measurement of extracted lipids from MITF^low^/AXL^high^ melanoma cells confirmed the CE-rich lipid profile and significant reduction of multiple CE species after avasimibe treatment (Figure 2(g) and Figure S5g). The mass spectrometry data identified cholesteryl oleate (CE 18 : 1) to be the dominant species. Together, these results indicate that LDs accumulated in MITF^low^/AXL^high^ melanoma contain an elevated level of unsaturated fatty acids in the form of triglyceride and CE.

### 2.3. Fatty Acid Uptake Is the Major Pathway for LD Accumulation in MITF^low^/AXL^high^ Melanoma

**Figure 3:**
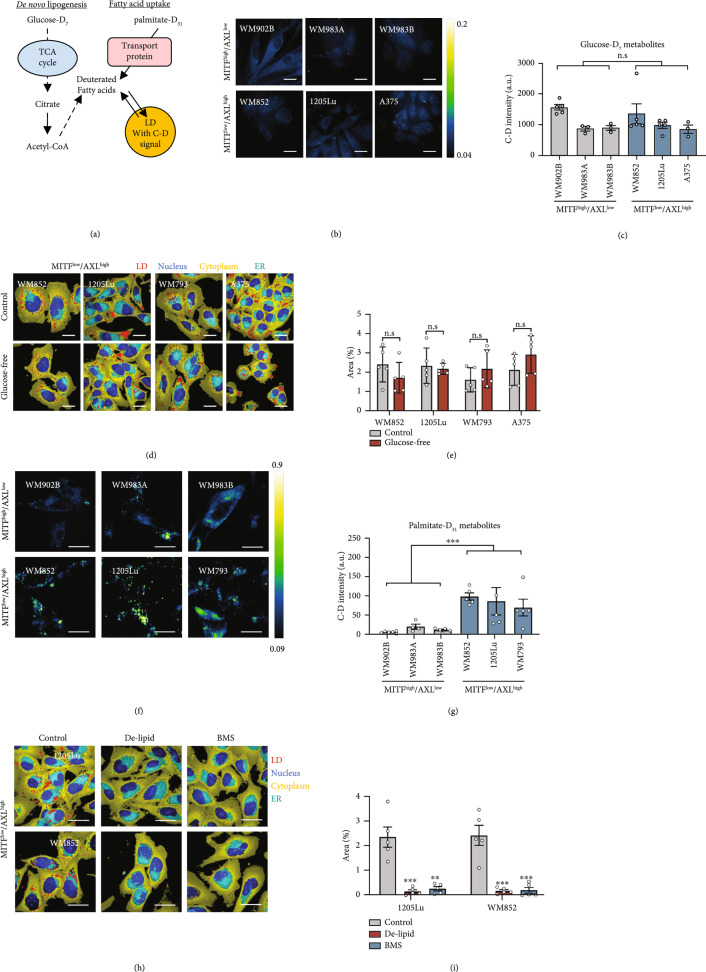
Fatty acid uptake is the major route for LD accumulation in MITF^low^/AXL^high^ melanoma. (a) A schematic of lipid sources contributing to LD synthesis and deuterated metabolite tracking method. (b) Representative SRS images in the C-D region (2127 cm^-1^) of MITF^high^/AXL^low^ and MITF^low^/AXL^high^ cells cultured with glucose-D_7_ containing media for 2 days. (c) Quantification of SRS intensity at 2127 cm^-1^ in cells (n=3-5 fields of view). (d) Representative phasor output from SRS images of MITF^low^/AXL^high^ cells, cultured in normal media as control and in glucose-free media (2 days). (e) Percentage area of LDs in MITF^low^/AXL^high^ cells (n=5 fields of view). (f) Representative SRS images in the C-D region (2127 cm^-1^) of WM902B, WM983A, WM983B, WM852, 1205Lu, and WM793, cultured with palmitate-D_31_ containing media (100 *μ*M, 6 hours). (g) Quantification of SRS intensity at 2127 cm^-1^ for C-D positive LDs (n=4-6 fields of view). (h) Representative phasor output from SRS images of 1205Lu and WM793, cultured in normal media as control, in delipidized serum media (2 days), and treated with BMS (50 *μ*M, 1 day). (i) Percentage area of LDs in 1205Lu and WM793 (n=5 fields of view). Scale bars, 10 *μ*m. Data represent mean±SD. ${}^{*}p<0.05$, ${}^{**}p<0.01$, and ${}^{***}p<0.001$. n.s.: not significant.

There are two major routes for lipid accumulation in mammalian cells: one is de novo lipogenesis and the other is fatty acid uptake through transporter proteins (Figure 3(a)). In *de novo* lipogenesis, glucose typically serves as the precursor, from which the acetyl-CoA is generated and used for lipid synthesis. Fatty acid uptake is facilitated by various fatty acid transporters, such as FABP4, CD36, and FATPs. In both cases, excess fatty acids are stored in LDs. To understand how MITF^low^/AXL^high^ melanoma cells obtain the lipids stored in LDs, we performed SRS imaging of deuterated metabolites in melanoma cells to evaluate their glucose-derived lipogenesis and fatty acid uptake activities. As SRS imaging of glucose-D_7_ has been used to track the de novo lipogenesis activity of cancer cells [25], we cultured MITF^high^/AXL^low^ and MITF^low^/AXL^high^ melanoma cells with a glucose-D_7_-containing medium. SRS imaging in the C-D vibration at 2127 cm^-1^ was performed to visualize glucose-D_7_-derived metabolites (Figure 3(b)). Interestingly, a weak signal was observed from the cytoplasm in both MITF^high^/AXL^low^ and MITF^low^/AXL^high^ melanoma cells (Figure 3(b)). No obvious C-D-derived droplet structures were observed in both groups. The quantification of the intracellular C-D signal showed no difference between the two groups (Figure 3(c)) up to 72 hours (Figure S6a, b), indicating no significant difference in glucose-derived metabolites. To further evaluate the contribution of de novo lipogenesis to LD accumulation in MITF^low^/AXL^high^ melanoma cells, we cultured the cells in a glucose-free medium. We then performed hyperspectral SRS imaging in C-H vibrations and quantified the LD area fraction by phasor analysis. The LD levels remained the same even without supplement of glucose (Figures 3(d) and 3(e)), indicating minimum contribution from *de novo* lipogenesis.

To evaluate the fatty acid uptake activity, palmitate-D_31_ was supplemented into the culture medium. SRS imaging at 2127 cm^-1^ clearly showed LDs incorporated with deuterated fatty acids, especially in MITF^low^/AXL^high^ melanoma cells (Figure 3(f)). To quantify LDs with the C-D signal, we performed particle analysis and measured the C-D intensity per cell area in each group. MITF^low^/AXL^high^ melanoma cells showed a significantly higher C-D signal than MITF^high^/AXL^low^ melanoma cells (Figure 3(g) and Figure S6c, d), indicating high palmitate uptake activity in these cells. Similarly, when oleate-D_34_ was supplemented into the culture medium, MITF^low^/AXL^high^ cells showed higher oleate uptake activity than MITF^high^/AXL^low^ cells (Figure S6e, f). To further validate that fatty acid uptake is the major source of LD accumulation in these cells, we suppressed fatty acid uptake by removing extracellular fatty acids using a delipidized serum. Hyperspectral SRS imaging at C-H vibrations and phasor analysis showed a significant reduction in the number of LDs when fatty acid availability became limited (Figures 3(h) and 3(i)). Similarly, when fatty acid uptake was suppressed by treating the cells with an inhibitor (BMS309403) targeting fatty acid-binding protein 4 (FABP4), a fatty acid transporter, the number of LDs reduced significantly (Figures 3(h) and 3(i)). Collectively, these data show that fatty acid uptake is the major route for LD accumulation in MITF^low^/AXL^high^ melanoma.

To validate these findings in clinical samples, we analyzed the expression of genes related to lipogenesis and fatty acid uptake in human melanoma samples from TCGA divided into primary and metastatic melanoma (Figure S6g). On average, MITF expression is reduced whereas the AXL expression level increased in the metastatic melanoma group, validating that MITF^low^/AXL^high^ melanoma cells represent metastatic melanoma more closely. Importantly, the rate-limiting enzyme in de novo lipogenesis, fatty acid synthase (FASN), showed no difference between primary and metastatic melanomas (Figure S6g), which is consistent with the SRS imaging results for evaluating lipogenesis activity shown in Figures 3(b) and 3(c). However, fatty acid transporters such as CD36 and FABP4 were upregulated in metastatic melanoma compared to primary melanoma (Figure S6g), suggesting increased fatty acid uptake activity. Since metastatic melanoma is a heterogeneous disease, we stratified metastatic melanoma samples into MITF^high^/AXL^low^ and MITF^low^/AXL^high^ groups based on their relative MITF and AXL levels. Within the metastatic melanoma samples, there was a clear difference in the expression of lipid metabolism-related genes based on MITF/AXL status (Figure S6h). The metastatic MITF^low^/AXL^high^ group showed upregulation of multiple fatty acid transporters and downregulation of genes involved in lipogenesis compared to the MITF^low^/AXL^high^ group. Together, while lipid *de novo* synthesis activity is at the same level, fatty acid uptake activity is found to be elevated in human metastatic melanoma.

### 2.4. Fatty Acid Sapienate Significantly Promotes Cell Migration

**Figure 4:**
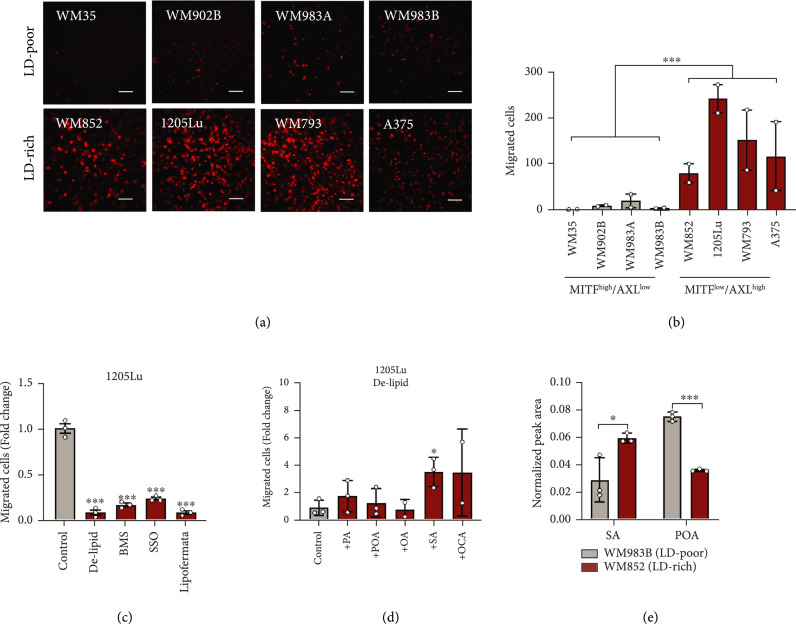
Fatty acid sapienate significantly promotes cell migration. (a) Images and (b) quantification of migrated MITF^high^/AXL^low^ and MITF^low^/AXL^high^ melanoma cells. n=2 biological replicates. (c) Quantification of migrated 1205Lu precultured with delipidized medium or pretreated with BMS309403 (BMS, 50 *μ*M, 1 day), sulfosuccinimidyl oleate (SSO, 50 *μ*M, 1 day), and lipofermata (10 *μ*M, 1 day). Control group was used for normalization. n=3 biological replicates. (d) Quantification of migrated 1205Lu precultured with delipidized serum media (1 day) and supplemented with ethanol as control and fatty acids (20 *μ*M, 12 hours) as indicated. Control group was used for normalization. n=3 biological replicates. (e) Quantification of normalized peak areas of sapienate and palmitoleate presented in WM852 and WM983B. n=3 biological replicates. Scale bars, 50 *μ*m. Data represent mean±SEM. ${}^{*}p<0.05$ and ${}^{***}p<0.001$.

To explore the relationship between LD accumulation and melanoma aggressiveness, we classified the melanoma cell lines into two groups based on their LD accumulation level (Table S1). The LD-rich melanoma cells showed significantly higher migration capacity than LD-poor melanoma cells (Figures 4(a) and 4(b)). Since LD accumulation mainly comes from fatty acid uptake (Figure 3), we examined the migration capacity of LD-rich cells after inhibition of fatty acid uptake to deplete LDs. When the LD accumulation was suppressed by depleting extracellular lipids with delipidized serum (Figures 3(f) and 3(g)), the number of migrated cells reduced significantly (Figure 4(c) and Figure S7a). Moreover, when the LD-rich cells were treated with various fatty acid transporter inhibitors, including BMS309403 (FABP inhibitor), sulfosuccinimidyl oleate (CD36 inhibitor), and lipofermata (FATP inhibitor), migration capacity significantly reduced (Figure 4(c) and Figure S7a). These results indicate that LD accumulation, driven by fatty acid uptake, contributes to the enhanced melanoma migration potential.

Next, we asked whether certain fatty acids play a more important role in promoting cell migration than others. We evaluated the migration rescue capability of various fatty acids by supplementing them into the delipidized serum (Figure 4(d) and Figure S7b). Palmitate (PA), palmitoleate (POA), and oleate (OA) did not rescue cell migration in the delipidized serum (Figure 4(d) and Figure S7b). Intriguingly, supplementation of sapienate (SA) and its elongated form, octadecenoate (OCA), rescued cell migration significantly (Figure 4(d) and Figure S7b), indicating an essential role of these fatty acids in cell migration. To validate the presence of sapienate in LD-rich cells, we measured and compared sapienate levels between LD-rich and LD-poor melanomas. Both sapienate (cis-6-hexadecenoate) and palmitoleate (cis-9-hexadecenoate) are monounsaturated fatty acids that enhance the unsaturation degree in cells, but the positions of the double bond are different. To specifically measure sapienate, we applied GC/MS to separate and detect sapienate and palmitoleate. Sapienate shows a peak at an earlier retention time than palmitoleate (Figure S7c), and this protocol allows us to separate these two fatty acids to obtain the amount of sapienate in the sample. By fatty acid methyl ester (FAME) analysis, we extracted free fatty acids from LD-rich and LD-poor melanoma cells. On the basis of the GC/MS analysis, the LD-rich melanoma is found to contain a much higher amount of sapienate than the LD-poor melanoma (Figure 4(e) and Figure S7d). Interestingly, the palmitoleate content shows the opposite trend, with a higher amount in LD-poor melanoma than in LD-rich melanoma (Figure 4(e)). Together, these results suggest that sapienate is an essential fatty acid in LD-rich melanoma and has an important functional role in enhancing cell migration.

### 2.5. Inhibition of FADS2 Suppresses Human Melanoma Migration *In Vitro* and Metastasis *In Vivo*

**Figure 5:**
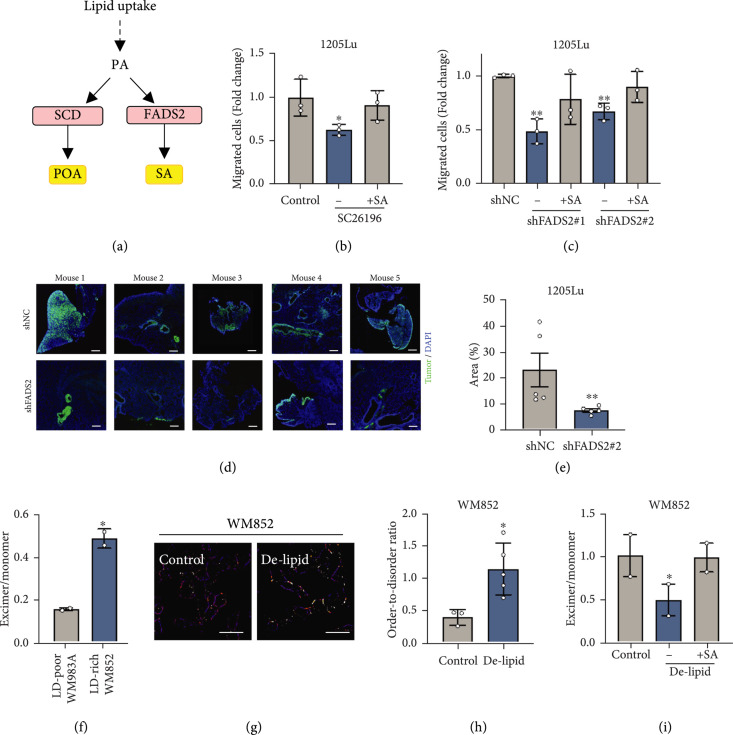
Inhibition of FADS2 suppresses melanoma migration and metastasis through modulation of membrane fluidity. (a) A schematic of two major fatty acid desaturases and their products. (b) Quantification of migrated 1205Lu treated with DMSO as a control, with SC26196 (50 *μ*M, 2 days), and with SC26196 with supplement of sapienate (20 *μ*M, 1 day during SC26196 treatment). Control group was used for normalization. n=3 biological replicates. (c) Quantification of migrated 1205Lu stabling expressing control shRNA (1205Lu-shNC) or FADS2 shRNAs (1205Lu-shFADS2 #1 and #2). 1205Lu-expressing FADS2 shRNAs were cultured in sapienate supplemented medium (20 *μ*M, 1 day) for testing the rescue capability. n=3 biological replicates. (d) Fluorescence images of lung tissues from mice 46 days after injected with 1205Lu-shNC or 1205Lu-shFADS2 #1 expressing GFP into the tail vein. Scale bars: 200 *μ*m. (e) Quantification of percentage area that tumors occupy in lung sections. Data represent mean±SEM. n=5 mice per group. (f) The ratio between excimer and monomer of membrane fluidity probe (pyrenedecanoic acid, PDA) in WM983A and WM852. n=2 biological replicates. (g) Ratiometric images of membrane fluidity probe (di-4-ANESPPDHQ) in WM852 cultured in a normal medium as control and delipidized serum medium. Scale bars: 50 *μ*m. (h) Quantification of order-to-disorder ratio obtained from (g). n=3-5 fields of view. (i) The ratio between excimer and monomer of PDA in WM852 cultured with a normal medium as control, with delipidized serum medium, and with delipidized serum medium with sapienate supplement (50 *μ*M, 1 day). n=2 biological replicates. Data represent mean±SEM. ${}^{*}p<0.05$ and ${}^{**}p<0.01$.

The opposite trends of sapienate and palmitoleate levels in LD-poor and LD-rich melanoma (Figure 4(e)) triggered us to investigate the role of sapienate metabolism in melanoma aggressiveness. Sapienate is synthesized from palmitate by FADS2, while palmitoleate is synthesized from palmitate by SCD (Figure 5(a)). A recent study indicates that these two desaturases can be complementary to each other to support cancer progression [26]. Importantly, our TCGA database analysis indicates that while SCD is expressed at similar levels, FADS2 expression is significantly upregulated in metastatic melanoma compared to primary melanoma (Figure S8a). To further evaluate the importance of FADS2-mediated fatty acid desaturation in melanoma metastasis, we tested the impact of FADS2 inhibition on cell migration (Figures 5(b) and 5(c)). FADS2 was inhibited by an inhibitor, SC26196, or by shRNA (Figure S8b). After inhibition of FADS2 by SC26196, cell migration was suppressed 1.5-fold compared to the control group (Figure 5(b)). As additional evidence, FADS2 knockdown by shRNA resulted in reduced cell migration by up to 2-fold, depending on the degree of knockdown efficiency (Figure 5(c) and Figure S8b). Importantly, sapienate supplementation rescued the migration capacity of the cells with either FADS2 inhibition methods (Figures 5(b) and 5(c)), indicating that sapienate has a functional role in promoting melanoma cell migration. Furthermore, FADS2 knockdown suppressed cell invasion, another important property of cancer metastasis, by 2-fold (Figure S8c, d). To evaluate the role of FADS2 in metastasis, we used a mouse tail-vein model. Human melanoma cells stably expressing control shRNA or FADS2 shRNA were intravenously injected, and the amount of lung metastases was compared (Figure 5(d)). Mice injected with melanoma cells containing FADS2 knockdown developed fewer lung metastases compared to the mice injected with control melanoma cells (Figure 5(d)). FADS2 knockdown resulted in a 68% reduction in the fractional area of lung metastases compared to the control group (Figure 5(e)). When the other fatty acid desaturase, SCD1, was inhibited, the migration capacity of MITF^low^/AXL^high^ melanoma cells was not significantly changed (Figure S8e, f), suggesting that FADS2 is a specific target for suppressing melanoma metastasis. Together, these results indicate that FADS2 is a potential therapeutic target for suppressing melanoma metastasis.

### 2.6. Melanoma Does Not Depend on Fatty Acid Oxidation for Energy Production, Proliferation, or Migration

One major function of fatty acids is providing energy through *β*-oxidation [27]. To investigate whether elevated fatty acid uptake in LD-rich melanoma cells supports fatty acid oxidation, we performed extracellular flux analyses using a Seahorse XFe96 analyzer on melanoma cells treated with or without 4 *μ*M etomoxir to inhibit mitochondrial fatty acid oxidation (Figure S9a, b). There were no differences in total ATP generation between etomoxir-treated cells and control cells (Figure S9a). In more detail, we analyzed the energy metabolic status of the cells based on mitochondrial and glycolytic ATP generation (Figure S9b). There were no differences in mitochondrial or glycolytic ATP generation between etomoxir-treated cells and control cells; thus, no metabolic shift was observed. Consistent with this finding, the on-target 5 *μ*M etomoxir dose had no effect on LD-rich melanoma cell proliferation while the off-target 200 *μ*M etomoxir dose slightly reduced cell proliferation (Figure S9c, d). Although high doses of etomoxir are used ubiquitously, recent research shows that higher doses (>10 *μ*M) have off-target effects leading to oxidative phosphorylation suppression through Complex I inhibition, which is erroneously interpreted as a reduction in fatty acid oxidation [28, 29]. Because of this, caution must be taken when interpreting data using high concentrations of etomoxir to inhibit fatty acid oxidation. Furthermore, LD-rich melanoma cells treated with the on-target 5 *μ*M dose showed no significant change in the cell migration capacity compared to control cells (Figure S9e, f). Collectively, these results indicate that LDs in metastatic melanoma are not used for fatty acid oxidation in normal, nonstarved conditions.

### 2.7. Sapienate Modulates Membrane Fluidity

Since unsaturated fatty acids are known to increase membrane fluidity and increased membrane fluidity facilitates cell migration and cancer metastasis [30], we hypothesize that sapienate and its downstream metabolites modulate membrane fluidity. LD-rich melanoma cells show higher membrane fluidity than LD-poor melanoma cells (Figure 5(f)). Furthermore, when the fatty acid uptake was suppressed by culturing cells in delipidized serum, the membrane fluidity was reduced significantly by 2-fold (Figures 5(g) and 5(h)). Importantly, sapienate supplemented into the delipidized serum was sufficient to rescue this effect, restoring membrane fluidity to a level similar to the control group (Figure 5(i)). These results suggest that sapienate plays a role in regulating membrane composition and dynamics, which potentially promotes melanoma cell migration *in vitro* and tumor metastasis *in vivo*.

## 3. Discussion

Altered lipid metabolism is being increasingly recognized in aggressive cancers [31]. Cancer cells increase their reliance on de novo biosynthesis or exogenous fatty acid uptake. These lipid metabolic features provide biomolecules to sustain rapid proliferation and, at the same time, serve as an energy source during metabolic stress [27]. Among the many aspects of lipid metabolism, fatty acid uptake is shown to be elevated in cancers with high metastatic potential [32, 33]. From this point of view, the metabolic microenvironment of tumors, such as adjacent adipocytes, has been shown to supply lipids and fatty acids to support cancer progression and promote metastasis [17, 34, 35]. In addition to lipid degradation to promote cancer survival [36], fatty acids provide energy through fatty acid oxidation to enhance metastasis [37, 38]. One particular lipid species that actively contributes to cancer progression is cholesterol [39]. Increased cholesterol biosynthesis and uptake are elevated in aggressive cancers, and the mevalonate pathway, part of cholesterol synthesis, interacts closely with oncogenic pathways [40, 41]. Accumulation of CE in LDs is found in multiple types of cancer [39], which serves as a reservoir of excess cholesterol to maintain cholesterol synthesis and uptake. These studies highlight the importance of lipid metabolism during cancer progression.

Despite various efforts to understand lipid metabolic reprogramming in cancer, the alterations in composition, intracellular distribution, and dynamics of lipids remain understudied. Lipids are highly dynamic and complicated species with functional roles dependent on their distribution. For example, lipid composition changes the physical properties of the membrane, such as fluidity, protein localization and activity, cell adhesion, and migration [30]. LDs are specialized storage organelles receiving attention because of their multiple functions such as serving as an energy source [42], supporting membrane biosynthesis [43], protecting cells from lipotoxicity [44] and oxidative stress [45], maintaining ER homeostasis [46], and interactions with other organelles [47]. Analyzing the composition of LDs and their spatiotemporal dynamics in cancer may suggest connections between oncogenic events and metabolic reprogramming for the development of new therapeutic targets.

High-speed, high-resolution chemical imaging enabled by coherent Raman scattering microscopy offers an effective tool to understand the altered metabolism inside cancer cells [48]. By SRS imaging and Raman spectroscopic analysis of single LDs in human prostate cancer patient tissues and cells, accumulation of CE in aggressive cancer was discovered, and cholesterol esterification was demonstrated as a therapeutic target of metastatic prostate cancer [49]. Using single-cell hyperspectral SRS imaging of ovarian cancer, unsaturated lipid was identified as the metabolic signature of cancer stem cells, and inhibition of lipid desaturation can effectively eliminate cancer stem cells [50]. Enabled by the high-speed spectral acquisition by the multiplexed SRS imaging scheme, the high-content analysis using SRS imaging cytometry unveiled a metabolic response to stress in cancer cells, indicating LD-rich protrusions with high lipolysis and fatty acid oxidation activities to support cancer survival under starvation and chemotherapy [51]. Collectively, these studies demonstrate the capability of coherent Raman scattering microscopy in understanding metabolic alterations in cancer with subcellular resolution and temporal dynamics.

An intriguing question is why metastatic melanoma favors fatty acid uptake over de novo lipogenesis. One possible reason is that melanoma is developed in a lipid-rich local environment [17, 34]. Upregulation of several fatty acid transporter genes, such as FABP4 [33, 34] and CD36 [32, 52], has been reported in metastatic cancer. It was found that stress such as hypoxia upregulates FABP4 expression [33]. Following enhanced fatty acid uptake, fatty acid oxidation is found to play an important function in cancers. For example, fatty acid oxidation helps cancer cells to survive under metabolic stress [51]. In melanoma, fatty acid oxidation-dependent drug resistance has been reported [53]. Inhibition of fatty acid oxidation suppresses metastasis to local lymph nodes [37], indicating the importance of fatty acids in promoting local metastasis in melanoma. Intriguingly, blood-borne metastasis to the lungs was not suppressed by fatty acid oxidation inhibition [37], suggesting that other functions of fatty acids exist that promote melanoma metastasis beyond local lymph nodes. In this study, we identified another important function of fatty acids in the cell, which is through the regulation of membrane fluidity and posttranslational modifications of proteins.

A recent independent study by Du et al. showed increased *de novo* fatty acid synthesis in one of the primary melanoma cell lines that belongs to the differentiated type [54]. In their work, while the undifferentiated type showed much lower de novo fatty acid synthesis, it contained LDs with a much higher unsaturation level. Here, we identified increased fatty acid uptake activities that contribute to LD accumulation in multiple undifferentiated MITF^low^/AXL^high^ melanoma cell lines. Fatty acid uptake as the major source of LD accumulation, but not *de novo* lipogenesis, is further supported by the results showing no difference of the LD amount in metastatic melanoma after culturing in glucose-free medium (Figures 3(d) and 3(e)). It is exciting that both studies identified common metabolic phenotypes of metastatic melanoma, i.e., increased cholesteryl ester and unsaturation degree of lipids.

There are two major fatty acid monodesaturation pathways: SCD-mediated, generating palmitoleate, and FADS2-mediated, generating sapienate. SCD is essential for cancer cell survival, and its anticancer potential has been reported in multiple cancers [55]. However, not all cancer cells are responsive to SCD inhibition [31], and a recent study demonstrated FADS2 as an alternative desaturation pathway in SCD-independent cancer cells [26]. Unlike other fatty acids, sapienate is only found in humans and is a major component of human sebum [56]. Intriguingly, a recent study indicated that SCD expression is positively regulated by MITF [57]. Therefore, one potential mechanism of increased dependency on FADS2-mediated fatty acid desaturation in MITF^low^/AXL^high^ LD-rich melanoma cell lines could be a compensatory mechanism due to reduced MITF-regulated SCD. At the same time, a recent report revealed that FADS2-mediated sapienate metabolism is regulated by the mTOR-SREBP1/2 axis [58], providing another possibility for the regulatory mechanism of FADS2 in melanoma. Importantly, as the high migration and invasion capacities presented in the melanoma can be suppressed by FADS2 inhibition *in vitro* and *in vivo*, inhibition of FADS2-mediated fatty acid desaturation offers a therapeutic opportunity to treat metastatic melanoma.

In addition to serving as a ∆6 desaturase to generate sapienate from palmitate, FADS2 mediates desaturation to generate polyunsaturated fatty acids. In metastatic melanoma, this sapienate synthetic enzyme is a major contributor promoting cell migration. This function is supported by the capacity of sapienate to rescue cell migration capacity in the delipidized serum condition. Further, the higher amount of sapienate found in LD-rich melanoma compared to LD-poor melanoma suggests elevated sapienate synthesis in metastatic melanoma. Mechanistically, unsaturated lipids on the plasma membrane are known to promote cell mobility [30]. In line with this observation, the membrane fluidity is higher in LD-rich melanoma compared to LD-poor melanoma, and supplementation of sapienate increases membrane fluidity. Intriguingly, the supplementation of palmitate in delipidized serum slightly rescued migration capacity. It is likely that high FADS2 activity in MITF^low^/AXL^high^ melanoma cells converted the palmitate into sapienate, thus increasing the membrane fluidity. It would be important to test this hypothesis to unveil the functional roles of specific fatty acids in cancer cells. We note that unsaturated fatty acids in general would enhance the membrane fluidity. Therefore, this mechanism may not be specific to sapienate alone. Nevertheless, these results collectively indicate an essential function of sapienate in cancer migration.

In parallel to the reprogramming of fatty acid metabolism in metastatic melanoma, cholesterol metabolism also plays an essential role in promoting cell migration. A significant amount of CE in LDs of metastatic melanoma suggests a rewiring of cholesterol metabolism. CE is synthesized from cholesterol and fatty acids by SOAT protein (Figure S10a). With the increasing number of reports regarding the upregulation of SOAT-mediated cholesterol esterification in cancers [39], it is essential to understand the physiological meaning and function of CE accumulation in metastatic disease. Our results demonstrate that SOAT inhibition effectively suppresses melanoma migration (Figure S10b, c), indicating that cholesterol esterification is another potential target for metastatic melanoma. Based on the reduced LD accumulation observed in SOAT-inhibited melanoma (Figure S10d), it is likely that cholesterol esterification is connected to fatty acid uptake, storage, and utilization. Fatty acid is known to serve as a substrate for protein modification such as palmitoylation to regulate protein localization and activities. For example, Wnt family proteins require palmitoylation to be transported to the plasma membrane for secretion [59]. Indeed, when cholesterol esterification is inhibited by avasimibe, *β*-catenin is sequestered on the plasma membrane (Figure S10e), indicating inactivation of the Wnt/*β*-catenin pathway. Furthermore, the amount of membrane-bound Wnt5a reduced significantly by 3-fold when cholesterol esterification was inhibited by avasimibe (Figure S10f). A similar Wnt5a distribution was found in melanoma cells cultured with delipidized serum (Figure S10g), which limits fatty acid uptake. Although cholesterol itself can modify signaling molecules such as the hedgehog protein [60], we did not observe inactivation of the hedgehog pathway when cholesterol esterification is inhibited (Figure S10h). Together, these results indicate that the cholesterol metabolism plays an important role in melanoma metastasis, possibly mediated by a change of fatty acid availability for protein modification (Figure S10i). As Wnt5a is a robust marker of aggressive and metastatic melanoma [61], our finding of modulation of Wnt5a modification by reprogrammed lipid metabolism provides an essential mechanism of linking metabolism with signaling pathways.

Finally, the metabolic reprogramming from pigmentation to lipid accumulation identified in this study serves as a molecular signature for metastatic melanoma diagnosis. It was found that more eumelanin than pheomelanin is present in malignant melanoma compared to dysplastic nevi [6]. Using pump-probe imaging, the distribution of these two types of melanin was studied in human tissues, which provides a label-free imaging approach for melanoma diagnosis [8]. More recently, chemical analysis of melanin with its spatial distribution using pump-probe microscopy further demonstrated the potential of predicting the metastatic capability of melanoma [9]. Despite these advances, loss of MITF found in melanomas with an invasive phenotype [10] suggests loss of pigmentation, indicating a need to identify new molecular markers for detecting aggressive and invasive melanomas. In this study, by the integration of label-free SRS imaging of lipids and pump-probe imaging of pigments, we identified a metabolic reprogramming from pigmentation to lipid accumulation in metastatic melanoma. Such a multimodal imaging platform can potentially be used to diagnose primary melanoma and metastatic lesions in human tissue samples. Furthermore, with the continued development of a handheld SRS imaging device [62], *in vivo* SRS and pump-probe imaging promises a noninvasive approach for the clinical evaluation of melanoma.

## 4. Materials and Methods

### 4.1. Cell Lines and Cell Culture

Human melanoma cell lines (WM35, WM902B, WM983A, WM983B, 1205Lu, WM793, and A375) harboring the BRAF V600E mutation and human melanoma cell line (WM852) with wildtype BRAF were obtained from Dr. Meenard Herlyn (The Wistar Institute). Cells were grown in Dulbecco’s Modified Eagle’s Medium (DMEM, Invitrogen) media supplemented with fetal bovine serum (FBS) (10%), L-glutamine (2 mM), penicillin (1%), and streptomycin (1%). Human primary melanocytes (Life Technologies) were grown in Medium 254 (Gibco) with human melanocyte growth supplements (Gibco). Cell lines were incubated at 37°C in 5% CO_2_.

### 4.2. Chemicals

FBS was purchased from Life Technologies. Delipidized serum was purchased from Gemini Bio. CAY10566, BMS309403, sulfosuccinimidyl oleate, lipofermata, and etomoxir were purchased from Cayman Chemical. SC26196 was purchased from Santa Cruz. Avasimibe was purchased from Selleckchem. Glucose-D_7_, palmitic acid-D_31_, and oleic acid-D_34_ were purchased from Cambridge Isotope Laboratories. Palmitate, palmitoleate, oleate, cholesteryl oleate, and glyceryl trioleate were purchased from Sigma-Aldrich. Sapienate was purchased from Matreya. Octadecenoate (8(Z)-octadecenoic acid) was purchased from Larodan.

### 4.3. Time-Domain Multimodal SRS/Pump-Probe Imaging

SRS/pump-probe imaging was performed on a femtosecond SRS microscope. An ultrafast laser system with dual output at 80 MHz (InSight DeepSee, Spectra-Physics) provided pump and Stokes beams. In the SRS imaging scheme, 802 nm and 1045 nm beams serve as pump and Stokes beams, respectively, to be resonant with the C-H stretching vibration at 2899 cm^-1^. For the pump-probe imaging scheme, 802 nm and 1045 nm beams serve as probe and pump beams, respectively. For off-resonance imaging of the C-H stretching vibration, the pump beam was tuned to 845 nm, which corresponds to 2265 cm^-1^. In this imaging scheme, the pump-probe signal, generated by 1045 nm pump and 845 nm probe, becomes dominant because no endogenous Raman active biomolecules are presented in the samples. 1045 nm beam was modulated by an acoustooptic modulator (AOM, 1205-C, Isomet) at 2.2 MHz. Both beams were linearly polarized. A motorized translation stage was employed to scan the temporal delay between the two beams. Two beams were then sent into a home-built laser-scanning microscope. A 60x water immersion objective lens (NA=1.2, UPlanApo/IR, Olympus) was used to focus the light into the sample, and an oil condenser (NA=1.4, U-AAC, Olympus) was used to collect the signal. The stimulated Raman loss and pump-probe signals were detected by a photodiode, which was extracted with a digital lock-in amplifier (Zurich Instrument). The power of the tunable beam (802 nm and 845 nm) and the power of the 1045 nm beam at the specimen were maintained at ~10 mW and 5 to 25 mW, respectively. The images were acquired at 10 *μ*s pixel dwell time. No cell or tissue damage was observed during the imaging procedure.

### 4.4. Phasor Analysis for LD and Pigment Quantification

LDs and pigments were analyzed by phasor analysis using the ImageJ plugin [23]. The segmentation of LDs and pigments in the phasor space is shown in Figure S2. After the segmentation, the “Threshold” function was used to select droplets in the cells. The “Analyze Particles” function was then used to quantify the area fractions of droplets in the whole image area, then normalized to the cell number counted from the same image.

### 4.5. In Silico Analysis of Melanoma Using TCGA Datasets

The transcriptome dataset of melanoma tissues (TCGA-SKCM) obtained from TCGA was downloaded in Apr 2019 using the UCSC Xena browser. The patients were assigned to MITF^high^/AXL^low^ and MITF^low^/AXL^high^ groups based on the median expression level. Differentially expressed genes and analyses via Gene Set Enrichment Analysis were generated using NetworkAnalyst 3.0 [63]. Further enrichment analyses were performed in the included genes (FC>1.2, adjusted p<0.05) using Metascape [64].

### 4.6. Human Tissue Sample Preparation

Deidentified frozen specimens of human primary and metastatic melanoma tissues were purchased from IU Simon Comprehensive Cancer Center (Lafayette, IN). Informed written consent from all participants or next of kin was obtained prior to the research by the IU Simon Comprehensive Cancer Center. The study is approved by the Boston University Institutional Review Board (2256). The tissue samples were sliced using a cryostat at 10 to 20 *μ*m thickness. The time-domain multimodal SRS/pump-probe imaging was performed on these tissue slices without any processing or labeling.

### 4.7. Confocal Raman Spectral Measurement

Raman spectral analysis from individual LDs was performed using a commercial confocal Raman microscope (LabRAM HR Evolution, Horiba) at room temperature. A 15 mW (after the objective), 532 nm diode laser was used to excite the sample through a 40x water immersion objective (Apo LWD, 1.15 N.A., Nikon). The total data acquisition time was 60 s using the LabSpec 6 software. For each group, at least 10 spectra from individual LDs in different locations or cells were obtained. To analyze the spectrum, the background was removed manually based on the glass background profile, and peak intensity was measured using OriginPro. The calibration curve for CE percentage in LD was generated by measuring CE/triacylglyceride emulsion at various percentages and linearly correlate it with the 704 cm^-1^ peak (cholesterol rings) normalized with the 1445 cm^-1^ peak (CH_2_ bending vibration). The unsaturation degree of LD is determined by the peak intensity at 1654 cm^-1^ (C=C stretching vibration) normalized with the 1445 cm^-1^ peak.

### 4.8. Metabolite Extraction and Measurement by LC/MS and GC/MS

The cells were extracted using a modified Bligh-Dyer extraction [65]. To each sample, 0.4 mL of ice-cold 65% methanol was added and vortexed for 1 min. The samples were placed on ice, spiked with 1 *μ*g of C17 : 0 margaric acid as internal standard, then pulse vortexed to mix. Next, 0.25 mL of chloroform was added and the sample vortexed for an additional 5 minutes. The samples were centrifuged at 13,500 rpm for 5 minutes at 4°C. The bottom layer was removed for fatty acid analysis. The samples were dried using a rotary evaporation device at room temperature for 2 hours. The samples were then derivatized for GC/MS analysis. Each sample received 0.5 mL of 14% boron trifluoride solution (BF_3_) (Sigma-Aldrich #B1252) and reacted for 30 minutes at 60°C. Then, 0.25 mL of water and 1 mL of hexane were added. The samples were mixed, then dried with approximately 0.2 g of anhydrous sodium sulfate. The hexane layer was then collected and dried using a stream of nitrogen. The final derivatized sample was reconstituted in 0.1 mL of hexane for GC/MS analysis.

A Thermo Fisher TriPlus RSH auto sampler and Trace 1310 gas chromatography (GC) system coupled to a Thermo Fisher TSQ 8000 mass spectrometer (MS) was used to analyze FAME composition in each sample (Thermo Fisher Scientific, Waltham, MA). An Agilent Select FAME GC column (50 m×0.25 mm, film thickness 0.2 *μ*m) was used for the analysis (Agilent Technologies, Santa Clara, CA). The GC carrier gas was helium with a linear flow rate of 1.0 mL min^-1^. The programmed GC temperature gradient was as follows: time 0 minutes, 80°C, ramped to 175°C at a rate of 13°C min^-1^ with a 5-minute hold, then ramped to 245°C at a rate of 4°C min^-1^ with a 2-minute hold. The total run time was 38.3 minutes. The GC inlet was set to 250°C, and samples were injected in the split-less mode. The MS transfer line was set to 250°C, and the MS ion source was set to 250°C. MS data were collected in the selected ion monitoring (SIM) mode according to Table 1. All data were analyzed with Thermo Fisher Chromeleon (Version 7.2.9) software. A standard mixture of 37 FAME (Supelco, Sigma-Aldrich), sapienate (Larodan), and palmitoleate (Sigma-Aldrich) was used to confirm spectra and column retention times.

**Table 1:** SIM table for mass spectrometry analysis.

Name	RT	Window (min)	SIM mass
Palmitic (16 : 0) acid	11.9	1	227
Sapienic (16 : 1*Δ*6) acid	12.4	1	236
Palmitoleic (16 : 1*Δ*9) acid	12.6	1	236
Margaric (17 : 0) acid-ISTD	13.2	1	241

### 4.9. Isotope Metabolite Labeling Experiments

For tracking de novo lipogenesis, cells are cultured with glucose-D_7_ (4.68 g L^-1^) in the glucose-free complete medium for 48 hours. For tracking fatty acid uptake, 100 *μ*M of palmitic acid-D_31_ or oleic acid-D_34_ was supplemented in the complete medium and cultured for 6 hours. The pump laser was tuned to 855 nm to perform SRS imaging in the C-D region at 2127 cm^-1^. Pump and Stokes powers at the specimen were maintained at ~10 mW and 50 mW, respectively. To quantify the SRS intensity at the C-D region from LDs, the “Threshold” function in ImageJ was used to select LDs in the cells. Then, the total intensity of LDs was obtained after particle analysis and normalized by the number of cells in the corresponding image.

### 4.10. Hyperspectral SRS Imaging and Phasor Analysis

Hyperspectral SRS imaging was performed by a spectral focusing approach. Both beams were chirped by three 12.7 cm long SF57 glass rods with the Stokes beam chirped by additional glass rod to match the pulse durations of the both beams. The Raman shift was calibrated using DMSO. The images were acquired at 10 *μ*s pixel dwell time. No cell damage was observed during the imaging procedure. LDs were then analyzed by phasor analysis using the ImageJ plugin [23]. The cells were segmented into LD, ER, cytoplasm, and nucleus by spectral phasor.

### 4.11. Migration/Invasion Assay

0.2 million cells were seeded in the upper chamber of a 24-well plate insert (Corning™ Transwell™ 8 *μ*m Permeable Polycarbonate Membrane Inserts) in serum-free media. For the invasion assay, the insert was precoated with Matrigel (BD Biosciences). DMEM media supplemented with 20% FBS and 50 ng mL^-1^ EGF were added to the bottom chamber of the insert. For the treatment group, inhibitors or fatty acids were added to both the upper and bottom chambers. After 4.5 to 8 hours of migration, cells were fixed with either 10% formalin or 70% ethanol, and nonmigrated cells were removed with a cotton swab. Migrated cells were stained with 50 *μ*g mL^-1^ propidium iodide (Invitrogen) for 30 minutes at room temperature and then washed with phosphate-buffered saline 5 to 6 times. 8 to 10 random images were taken for each well using an FV3000 confocal microscope or a Nikon Eclipse E400 microscope. The number of migrated/invaded cells was quantified using ImageJ. Migrated cell count was normalized to the control group and represented as fold change from control unless indicated otherwise.

### 4.12. Knockdown Strategies

1205Lu cells were transfected with FADS2 targeting shRNA lentiviral particles (Applied Biological Materials, shFADS2 #1: CCGGCCACGGCAAGAACTCAAAGA-TCTCGAGATCTTTGAGTTCTTGCCGTGGTTTTTG, shFADS2 #2: CCGGCCAC CTGTCT-GTCTACAGAAACTCGAGTTTCTGTAGACAGACAGGTGGTTTTTG) following the protocol provided by the manufacturer. Scrambled shRNA lentiviral particles (shNC: GTCTCCACGCGCAGTACATTT) were used as a control. Stably transfected cells were selected with 1 *μ*g mL^-1^ puromycin. 1205Lu and WM852 cells were transfected with SOAT1 targeting shRNA lentiviral particles (Santa Cruz, sc-29624-V) following the protocol provided by the manufacturer. Stably transfected cells were selected with 1 *μ*g mL^-1^ puromycin.

### 4.13. Mouse Model and Fluorescence Imaging of Tissue Sections

All animal procedures were approved by Boston University IACUC (PROTO201800533). The mouse tail-vein injection model was used to study the development of metastatic cancer. 6-week-old male homozygous nude (Foxn1nu/Foxn1nu) mice obtained from the Jackson Laboratory were used. 1205Lu melanoma cells stabling expressing GFP (Applied Biological Materials) were transfected with lentivirus carrying negative control shRNA (1205Lu-shNC) or FADS2 targeting shRNA (1205Lu-shFADS2 #2). The cells were then prepared in sterile PBS in a completely monocellular suspension without clumps at $1\times 10^{6}\;\text{m}{\text{L}}^{-1}$ concentration. 100 *μ*L of cell suspension was injected slowly via the lateral tail vein of the anesthetized mouse, after which the bleeding was stopped by applying pressure to the puncture site with a dry piece of gauze. The lung tissues were collected 46 days after tumor inoculation and sliced using a cryostat at 10 to 20 *μ*m thickness. Then, the tissues were mounted with the antifade mounting medium with DAPI (Vector Laboratories). The tissues were imaged using an Olympus VS120 Automated Slide Scanning Microscope.

### 4.14. Immunofluorescence Staining and Imaging

Cells were fixed with 10% formalin for 15 min at room temperature. Cells were then incubated with anti-adipophilin (Millipore Sigma, 393A-1, 1 : 100), anti-FADS2 (Proteintech, 28034-1-AP, 1 : 200), anti-Gli1 (Abcam, ab49314, 1 : 200), anti-active *β*-catenin (Millipore Sigma, 05-665, 1 : 200), or anti-Wnt5a (Santa Cruz, sc-365370, 1 : 200). After incubation with the secondary antibody containing Alexa-Fluor 488, cells were mounted using the antifade mounting medium with DAPI (Vector Laboratories). Then, the cells were imaged under an FV3000 confocal microscope with a 60x oil objective. The fluorescence signal from the antibody was imaged with 488 nm excitation and 500-600 nm emission. DAPI signal was imaged with 405 nm excitation and 430-479 nm emission.

### 4.15. Membrane Fluidity Assay

Membrane fluidity was measured using a MarkerGene™ Membrane Fluidity Kit (Abcam) following the protocol provided by the manufacturer. Briefly, cells were grown on 96-well plates. After 24 to 48 hours, the cells were washed with PBS and incubated with 10 *μ*M of pyrenedecanoic acid (PDA) in a perfusion buffer containing 0.08% pluronic F127 for 20 minutes at 25°C in the dark. Then, the unincorporated PDA is removed by washing the cells with serum-free media twice. The incorporated PDA was measured by reading fluorescence signals at both 400 (monomer) and 460 nm (excimer) with excitation at 360 nm using a SpectraMax i3x Microplate Detection Platform (Molecular Devices). The membrane fluidity was represented as a ratio between excimer fluorescence and monomer fluorescence, in which a higher ratio indicates higher membrane fluidity.

### 4.16. Confocal Imaging of Membrane Fluidity

For imaging membrane fluidity, cells were grown on glass coverslips for 48 hours. Cells were stained with di-4-ANEPPDHQ (Thermo Fisher Scientific) following the protocol provided by the manufacturer. Briefly, 5 mM of di-4-ANNEPPDHQ stock solution in DMSO was diluted in serum-free DMEM (final concentration: 5 *μ*M). Cells were incubated with di-4-ANNEPPDHQ-containing DMEM for 30 minutes at 37°C in the dark. Then, cells were washed with DMEM and imaged using an FV3000 confocal microscope with excitation at 488 nm and emission at both 530-590 (ordered lipid) and 590-650 nm (disordered lipid). The images were analyzed using ImageJ to generate ratiometric results of ordered-to-disordered lipids.

### 4.17. Statistical Analysis

Statistical analysis was performed using OriginPro or Prism. For two-sample comparisons, data were first tested for normality. If the data is detectably non-Gaussian, a nonparametric Mann-Whitney U test was performed. Otherwise, a one-tailed Student’s t-test was performed. The probability of the null hypothesis p<0.05 was judged to be statistically significant.

## Data Availability

The data used to support the findings of this study are available from the corresponding authors upon request.

## Supplementary Materials

Supplementary Materials Figure S1: imaging LD and pigments in human melanoma cells grouped by MITF and AXL expressions. Table S1: summary of human melanoma cell lines used in the study. Figure S2: pigments and LDs can be separated by time-domain multimodal SRS/pump-probe imaging and phasor analysis. Figure S3: functional enrichment network analysis for human melanoma grouped based on MITF and AXL. Figure S4: hematoxylin and eosin (H&E) staining of adjacent slices used for multimodal SRS imaging in Figure 1(e). Figure S5. LDs in MITF^low^/AXL^high^ melanoma cells contain unsaturated fatty acids and CE. Figure S6. MITF^low^/AXL^high^ melanoma shows higher oleate uptake activity compared to MITF^high^/AXL^low^ melanoma. Figure S7: fatty acid sapienate significantly promotes cell migration. Figure S8: inhibition of FADS2 suppresses melanoma invasion. Figure S9: fatty acid *β*-oxidation is not the major source of energy in melanoma. Figure S10: inhibition of cholesterol esterification suppresses melanoma migration via regulation of Wnt/*β*-catenin pathway. Supplementary methods Table S2: GSEA of TCGA-SKCM cohort (MITF^high^/AXL^low^ vs. MITF^low^/AXL^high^) with KEGG, Reactome, and GO-BP subset. Table S3: GO subset enrichment analysis of DEGs between MITF^high^/AXL^low^ and MITF^low^/AXL^high^ patients.
