# Supplementary material for: Multimodal Metabolic Imaging Reveals Pigment Reduction and Lipid Accumulation in Metastatic Melanoma
Source: BME Front. 2021 Oct 8;2021:9860123. doi: 10.34133/2021/9860123 (PMC10521760; doi:10.34133/2021/9860123)
Supplement: Supplementary Materials — Figure S1: imaging LD and pigments in human melanoma cells grouped by MITF and AXL expressions. Table S1: summary of human melanoma cell lines used in the study. Figure S2: pigments and LDs can be separated by time-domain multimodal SRS/pump-probe imaging and phasor analysis. Figure S3: functional enrichment network analysis for human melanoma grouped based on MITF and AXL. Figure S4: hematoxylin and eosin (H&E) staining of adjacent slices used for multimodal SRS imaging in Figure 1(e). Figure S5. LDs in MITFlow/AXLhigh melanoma cells contain unsaturated fatty acids and CE. Figure S6. MITFlow/AXLhigh melanoma shows higher oleate uptake activity compared to MITFhigh/AXLlow melanoma. Figure S7: fatty acid sapienate significantly promotes cell migration. Figure S8: inhibition of FADS2 suppresses melanoma invasion. Figure S9: fatty acid β-oxidation is not the major source of energy in melanoma. Figure S10: inhibition of cholesterol esterification suppresses melanoma migration via regulation of Wnt/β-catenin pathway. Supplementary methods Table S2: GSEA of TCGA-SKCM cohort (MITFhigh/AXLlow vs. MITFlow/AXLhigh) with KEGG, Reactome, and GO-BP subset. Table S3: GO subset enrichment analysis of DEGs between MITFhigh/AXLlow and MITFlow/AXLhigh patients. [file 9860123.f1.zip › Melanoma Manuscript supp v18-BMEF.docx]

Supporting Information

Multimodal Metabolic Imaging Reveals Pigment Reduction and Lipid Accumulation in Metastatic Melanoma

Hyeon Jeong Lee^1†‡^, Zhicong Chen^1†^, Marianne Collard^2^, Fukai Chen^1^, Jiaji G Chen^2^, Muzhou Wu^2^, Rhoda M Alani^2*^, and Ji-Xin Cheng^1*^

^1^Photonics Center, Department of Electrical and Computer Engineering, Department of Biomedical Engineering, Boston University, Boston, MA 02215, USA

^2^Department of Dermatology, Boston University School of Medicine, Boston, MA 02118, USA

Corresponding authors: Rhoda M Alani; [alani@bu.edu](mailto:alani@bu.edu); Ji-Xin Cheng; [jxcheng@bu.edu](mailto:jxcheng@bu.edu)

^†^ These authors contributed equally to this work.

^‡^ Present address: College of Biomedical Engineering and Instrument Science, Key Laboratory for Biomedical Engineering of Ministry of Education, Zhejiang University, Hangzhou 310027, China

Figures S1 – S10, Table S1

Supplementary methods

Supplementary references

**Figure S1. Imaging LD and pigments in human melanoma cells grouped by MITF and AXL expressions.** (**a**) MITF mRNA levels in a panel of human melanoma cell lines. (**b**) AXL mRNA levels in the same panel of human melanoma cell lines. (**c**) Simultaneous SRS imaging in the C-H region and immunofluorescence analysis of adipophilin (green) in 1205Lu and WM852 cells. Scale bars, 10 µm. (**d**) Time-resolved pump-probe signals from droplets in HPM (human primary melanocyte), WM902B (MITF^high^/AXL^low^), and WM852 (MITF^low^/AXL^high^) cells. The curves were subtracted with the background generated from cross-phase modulation. No pump-probe signals were found in WM852 cells. (**e**) Time-resolved pump-probe signal from eumelanin derived from *Sepia Officinalis*.

**Table S1. Summary of human melanoma cell lines used in the study.**

**
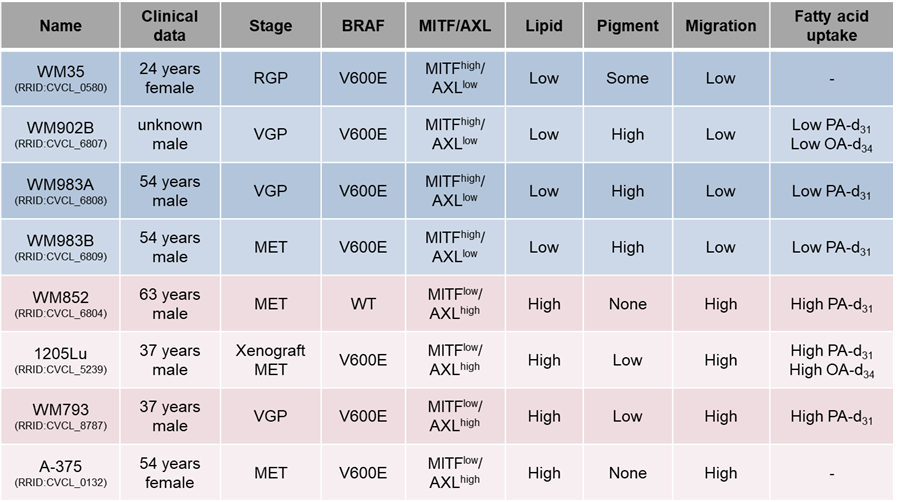
**

**Figure S2.** **Pigments and LDs can be separated by time-domain multimodal SRS/pump-probe imaging and phasor analysis.** (**a**) A schematic of SRS/pump-probe imaging. (**b**) A frame-by-frame time-resolved imaging was performed on MITF^low^/AXL^high^ and MITF^high^/AXL^low^ melanoma. (**c**) Time-resolved SRS and pump-probe signals from droplets in MITF^high^/AXL^low^ and MITF^low^/AXL^high^ melanoma. (**d**) A phasor plot of the time-resolved images of MITF^high^/AXL^low^ and MITF^low^/AXL^high^ melanoma. The regions representing pigments, background, lipid droplets and background are indicated. (**e**) Phasor output of multimodal SRS/pump-probe images. (**f**) Phasor output of multimodal SRS/pump-probe images from melanoma cell lines grouped based on MITF/AXL status. Scale bars, 20 µm. SRS: stimulated Raman scattering; LD: lipid droplets.

**Figure S3. Functional enrichment network analysis for human melanoma grouped based on MITF and AXL.** (**a**) Differentially expressed genes (DEGs) between MITF^low^/AXL^high^ and MITF^high^/AXL^low^ patients in TCGA-SKCM metastatic subgroup. (**b**) Enrichment analysis results for the DEG between MITF^low^/AXL^high^ and MITF^high^/AXL^low^ patients. Individual gene ontology terms with similar gene members and clustered by categories indicated by node colors and labeled using a representative member. Terms with a Kappa similarity score > 0.3 are connected by edges. Node size is proportional to enrichment p-value.

**Figure S4.** Hematoxylin and eosin (H&E) staining of adjacent slices used for multimodal SRS imaging in **fig. 1e**.

**Figure S5. LDs in MITF^low^/AXL^high^ melanoma cells contain unsaturated fatty acids and CE.** (**a**) Representative Raman spectra of palmitic acid, oleic acid, and linoleic acid. The peak representing C=C bond is highlighted in grey. (**b**) Calibration curve for quantification of unsaturation degree based on the number of C=C bonds, generated by linear fitting of height ratio between the peak at 1654 cm^-1^ (I_1654_) and the peak at 1445 cm^-1^ (I_1445_). I_1654_/I_1445_ = 0.788 × number of C=C. (**c**) Quantification of triacylglycerol species of lipids extracted from MITF^low^/AXL^high^ melanoma measured by MRM profiling. Data represent mean ± SEM (n = 3). (**d**) Representative Raman spectra of CE and triacylglycerol emulsions with five different molar percentages of CE, ranging from 0% to 100%. Emulsions are mixtures of cholesteryl oleate and glyceryl trioleate. (**e**) Calibration curve for quantification of molar percentage of CE out of total lipid, generated by linear fitting of height ratio between the peak at 702 cm^-1^ (I_702_) and the peak at 1445 cm^-1^ (I_1445_). I_702_/I_1445_ = 0.00353 × CE percentage. Data adopted from ref ^[1]^. (**f**) Quantification of CE percentage in lipids extracted from MITF^low^/AXL^high^ melanoma measured by MRM profiling. Data represent mean ± SEM (n = 3). (**g**) Mass spectra of lipids extracted from 1205Lu cells treated with DMOS as control and avasimibe (10 µM, 2 days). m/z 614.6, m/z 640.6, m/z 642.6, m/z 668.6, and m/z 670.7 stand for cholesteryl myristate (C14:0), cholesteryl palmitoleate (C16:1), cholesteryl palmitate (C16:0), cholesteryl oleate (C18:1), and cholesteryl stearate (C18:0), respectively. The spectral intensity shown in (**a**) and (**d**) was normalized by the CH_2_ bending band at 1445 cm^-1^.

**Figure S6. MITF^low^/AXL^high^ melanoma shows higher oleate uptake activity compared to MITF^high^/AXL^low^ melanoma.** (**a**) Representative SRS images in the C-D region (2127 cm^-1^) of MITF^high^/AXL^low^ and MITF^low^/AXL^high^ cells cultured with glucose-D_7_ containing media for 72 hours. (**b**) Quantification of SRS intensity at 2127 cm^-1^ in cells (n = 3 - 5 fields of view, mean ± SEM). (**c**) Representative SRS images in the C-D region (2127 cm^-1^) of MITF^high^/AXL^low^ and MITF^low^/AXL^high^ cells cultured with cultured with palmitate-D_31_ containing media (25 µM, 24 hours). (**d**) Quantification of SRS intensity at 2127 cm^-1^ for C-D positive LDs. (n = 3 - 5 fields of view, mean ± SEM). (**e**) Representative SRS images in the C-D (2127 cm^-1^) and C-H (2899 cm^-1^) regions of WM902B and 1205Lu, cultured with oleate-D_34_ containing media (100 µM, 6 hours). (**f**) Quantification of SRS intensity at 2127 cm^-1^ from C-D positive LDs. (**g**) Relative mRNA expression levels of MITF, AXL, FASN, CD36 and FABP4 in primary and metastatic melanoma from TCGA-SKCM database. N = 103 for primary, and 368 for metastatic. (**h**) Differences in expression levels of genes related to lipid metabolism between MITF^low^/AXL^high^ and MITF^high^/AXL^low^ metastatic melanoma from TCGA-SKCM database. Scale bars, 10 µm. Data represent mean ± SD, unless indicated otherwise. *: p < 0.05, **: p < 0.01, ***: p < 0.001.

**Figure S7. Fatty acid sapienate significantly promotes cell migration.** (**a**) Images of migrated 1205Lu pre-cultured with de-lipidized medium or pre-treated with BMS309403 (BMS, 50 µM, 1 day), sulfosuccinimidyl oleate (SSO, 50 µM, 1 day) and lipofermata (10 µM, 1 day). (**b**) Images of migrated 1205Lu pre-cultured with de-lipidized serum media (1 day) and supplemented with ethanol as control and fatty acids (20 µM, 12 hours) as indicated. Control group was used for normalization. Scale bars, 50 µm. (**c**) Detection of sapienate (SA) and palmitoleate (POA) by GC/MS. 16:0 Hexadecanoic acid was used as an internal control. (**d**) Mass spectra of lipids extracted from WM852 and WM983B. The peaks at 12.38 min and at 12.61 min stand for sapienate (SA) and palmitoleate (POA), respectively.

**Figure S8. Inhibition of FADS2 suppresses melanoma invasion.** (**a**) Relative mRNA expression levels of SCD and FADS2 in primary and metastatic melanoma from TCGA-SKCM database. (**b**) Relative FADS2 mRNA levels in 1205Lu stabling expressing control shRNA (shNC) or FADS2 shRNAs (shFADS2 #1 and #2). (**c**) Images and (**d**) quantification of invaded 1205Lu cells stabling expression shNC and shFADS2. (**e**) Images and (**f**) quantification of migrated 1205Lu cells treated with DMSO as control and CAY10566 (50 µM, 2 days). Scale bars, 50 µm. Data represent mean ± SEM. *: p < 0.05, **: p < 0.01, n.s.: not significant.

**Figure S9. Fatty acid β-oxidation is not the major source of energy in melanoma.** (**a**) ATP production in LD-poor and LD-rich melanoma cells treated with ethanol as control and 4 μM etomoxir. (**b**) A plot of mitochondrial ATP versus glycolysis ATP productions in melanoma cells treated with ethanol as control and 4 μM etomoxir. (**c**) Brightfield images of melanoma cells treated with ethanol as control, 5 μM etomoxir, and 200 μM etomoxir. Scale bar: 50 µm. (**d**) Quantification of cell proliferation in melanoma cells. (**e**) Images and (**f**) quantification of migrated 1205Lu and A375 pre-treated with ethanol as control and 5 μM etomoxir. Scale bar: 25 µm. Data present mean ± SD. *: p < 0.05, n.s.: not significant.

**Figure S10. Inhibition of cholesterol esterification suppresses melanoma migration via regulation of Wnt/β-catenin pathway.** (**a**) A schematic of cholesterol esterification process mediated by SOAT. (**b**) Images and quantification of migrated melanoma cells treated with DMSO as control, 10 µM avasimibe and 20 µM avasimibe for 2 days. Scale bars, 50 µm. (**c**) Images and quantification of migrated WM852 expressing shNC and shSOAT1. Scale bars, 50 µm. Data represent mean ± SEM. (**d**) Representative SRS images in the C-H region (2899 cm^-1^) and quantification of percent area of LDs in 1205Lu treated with DMSO as control, avasimibe (10 µM, 2 days), 1205Lu expressing negative control shRNA (shNC), and SOAT1 shRNA (shSOAT1). Scale bars: 10 µm. (**e**) Fluorescence images of immunostaining active β-catenin and quantification of membrane bound β-catenin in 1205Lu treated with DMSO as control and avasimibe (10 µM, 2 days). Arrows indicate membrane sequestered β-catenin. Scale bars, 50 µm. (**f**) Fluorescence images of immunostaining Wnt5a and quantification of membrane bound Wnt5a in 1205Lu treated with DMSO as control and avasimibe (10 µM, 2 days). Arrows indicate membrane bound Wnt5a. (**g**) Fluorescence images of immunostaining Wnt5a and quantifiction of membrane bound Wnt5a in 1205Lu cultured with de-lipidized serum for 2 days. Arrows indicate membrane bound Wnt5a. (**h**) Fluorescence images of immunostaining Gli1 and quantification of nuclear Gli1 in 1205Lu treated with DMSO as control and avasimibe (10 µM, 2 days). (**i**) Proposed molecular mechanism of cholesterol esterification modulating Wnt palmitoylation. Scale bars, 25 µm unless indicated otherwise. Data represent mean ± SD unless indicated otherwise. n.s.: not significant, *: p < 0.05, **: p < 0.01, ***: p < 0.001.

**Supplementary methods**

**RT-qPCR.** RNA was isolated following the RNeasy Plus Mini Kit protocol (Qiagen). RNA concentration was quantified using the NanoDrop™ (Thermo Fisher Scientific), and 1 μg RNA was reverse transcribed and amplified using the Superscript™ III First-Strand Synthesis System (Thermo Fisher Scientific). Resultant cDNA (1μL) was added to primer working solutions (IQ™ SYBER^®^ Green Supermix, UltraPure™ Distilled Water, and forward and reverse primer mix) for each well in the 96-well qPCR plate. Amplification and quantification were performed using the StepOnePlus™ Real-Time PCR System (Thermo Fisher Scientific). All reactions were performed in triplicate, using GAPDH as an internal control. Results were quantified as Ct values, which represent the threshold cycle of PCR at which the amplified product is first detected, and expressed as the ratio of target/control (relative gene expression) using the 2^−ΔΔCt^ method.

**Primer sequences used for RT-qPCR.**

| **mRNA Target** | **Forward Sequence (5’ -> 3’)** | **Reverse Sequence (5’ -> 3’)** |
| --- | --- | --- |
| MITF | GGAAATCTTGGGCTTGATGGA | CCCGAGACAGGCAACGTATT |
| AXL | CCAGGACACCCCAGAGGTGCTAAT | TGGTGGACTGGCTGTGCTTGC |
| FADS2 | GACCACGGCAAGAACTCAAAG | GAGGGTAGGAATCC |
| SOAT1 | CCACTGGTCCAGATGAGTTTAG | GGGAACATGCAGAGTACCTTT |
| GAPDH | TGGTATCGTGGAAGGACTC | AGTAGAGGCAGGGATGATG |

**Multiple reaction monitoring (MRM) profiling.** Lipids were extracted using the Bligh & Dyer (1959) method. The dried lipid extracts were diluted into were diluted in 200 μL injection solvent (acetonitrile/ methanol/ammonium acetate 300 mM 3:6.65:0.35 [v/v]) to obtain a stock solution. The stock solution was further diluted 50x into injection solvent spiked with 0.1 ng/µL of Equisplash Lipidomics (Avanti Polar Lipids # 330731) for sample injection. The MRM-profiling methods and instrumentation used were recently described by Lima et al. (2018) and Dipali et al. (2019). Mass spectrometry was acquired by flow-injection (no chromatographic separation) from 8 μL of the diluted lipid extract stock solution delivered using a micro-autosampler (G1377A) to the ESI source of an Agilent 6410 triple quadrupole mass spectrometer (Agilent Technologies, Santa Clara, CA, USA). A capillary pump was connected to the autosampler and operated at a flow rate of 7 μL/min and pressure of 100 bar. Capillary voltage on the instrument was 5 kV and the gas flow 5.1 L/min at 300 °C.

**Extracellular flux analysis.** Etomoxir was dissolved in sterile water to a final stock concentration of 2 mM. The Etomoxir stock solution was aliquoted and stored at -20°C. Fresh Etomoxir aliquots were used for each treatment, and treatments were refreshed every 2-3 days. Cells (15,000 cells/well) were plated on an XF96 polystyrene cell culture microplate (Seahorse®, Agilent). The following day, cells were washed and incubated in XF DMEM pH 7.4 assay medium supplemented with 10 mM glucose, 1 mM pyruvate, and 2 mM glutamine (Seahorse®, Agilent) in a CO_2_ free incubator for 1 hour. The plate was inserted into the Seahorse Xfe96 Extracellular Flux Analyzer, and injections of etomoxir (4 μM final concentration), Oligomycin (1.5 μM final concentration), and Antimycin A (1 μM final concentration) were sequentially added to assess the role of mitochondrial fatty acid oxidation on ATP generation. Simultaneous oxygen consumption rate (OCR) and extracellular acidification rate (ECAR) measurements were obtained every 3 minutes; a minimum of three measurements was acquired after each injection. OCR (pmol O_2_ min^-1^) and ECAR (mpH min^-1^) values were normalized to DNA content (ng μL^-1^) measured using the PicoGreen™ Assay. The Seahorse® Wave V2.6 software was used for data analysis.

**Supplementary References**

[1] H. J. Lee, J. Li, R. E. Vickman, J. Li, R. Liu, A. C. Durkes, B. D. Elzey, S. Yue, X. Liu, T. L. Ratliff, J. X. Cheng, *Mol. Cancer Res.* **2018**, *16*, 974.
